# Design of a fault-tolerant control system for a centrifugal pump-based level control system for sensor faults

**DOI:** 10.1038/s41598-026-42361-x

**Published:** 2026-03-19

**Authors:** Muhammad Irfan, Arslan Ahmed Amin, Saba Waseem, Saifur Rahman, Hatim Alwadie, Saleh Al Dawsari

**Affiliations:** 1 Electrical Engineering Department, College of Engineering, Najran University, 61441 Najran, Saudi Arabia; 2 Department of Electrical Engineering, FAST National University of Computer and Emerging Sciences, Chiniot Faisalabad Campus, Punjab, 35400 Pakistan; 3 School of Engineering, Cardiff University, Cardiff, CF24 3AA UK

**Keywords:** Active fault-tolerant control, Centrifugal pumps, Industrial process loops, Level control, PID controller, Sensor faults, Energy science and technology, Engineering, Mathematics and computing

## Abstract

Industrial activities rely on centrifugal pumps for fluid movement in numerous processes, including water treatment plants and chemical processing plants. These pumps must be used to precisely transfer the specific fluid level in tanks in order for operations to be stable and effective. Nevertheless, typical Proportional Integral Derivative (PID) controllers have the potential to deal with these types of problems, and the feedback measurement is based on sensors. Also, in industrial process control systems, where exact information is vital for maintaining stability and performance across applications, sensor faults are a significant challenge to address. Any issue with the level-measuring sensor could cause unnecessary downtime and incorrect production, which could have a big impact on the control loop. For such systems, this paper suggests a Fault-Tolerant Control (FTC) approach that actively estimates faulty sensor data in real-time using a Fault Detection and Isolation (FDI) estimator. The FDI unit of system estimates a value based on data from other operating sensors in the case of one sensor fault. The results show that the suggested strategy is an effective approach to keep the pump running even if the sensor fails. The control loop is based on an estimate from the FDI unit; thus, it continues to operate even if a sensor fails. Given the numerous industrial applications for centrifugal pumps, the suggested approach guarantees improved efficiency and fault tolerance.

## Introduction

### Fault-tolerant control overview

The fault-tolerant control systems (FTCS) goal is to keep the system running smoothly even if a component fails. Architectural differences and features define two major categories of FTCS: active and passive^1^. Active fault-tolerant control systems (AFTCS) identify and isolate faults using a fault detection and isolation (FDI) unit. The first part of AFTCS, FDI, estimates values for system parameters using an observer model; these values are matched with the real sensor inputs. One compares the calculated difference with a set threshold and determines if the component is healthy or faulty. To adjust to new circumstances following fault isolation, controller reconfiguration is performed^2^. The observer design process, which is discussed below, is thoroughly explained by Wang et al.^3^ and Nise^4^.

1 $$\dot{x} = Ax + Bu$$

2 $$y = Cx + Du$$

3 $$\dot{\bar{x}} = A\bar{x} + Bu$$

4 $$\bar{y} = C\bar{x} + Du$$

5 $$(\dot{\bar{x}} - \dot{x}) = A(\bar{x} - x)$$

6 $$(\bar{y} - y) = C(\bar{x} - x)$$

7 $$\dot{\bar{x}} = A\bar{x} + Bu + L(\bar{y} - y)$$

8 $$\dot{\bar{x}} - \dot{x} = A(\bar{x} - x) + L(\bar{y} - y)$$

9 $$\dot{\overline{x} }-\dot{x}=(A+LC)\left(\overline{x }-x\right)$$

10 $${\dot{e}}_{x}=\left(A+LC\right){e}_{x}$$

11 $$\left(\overline{y }-y\right)=C{e}_{x}$$

**Fig. 1:**
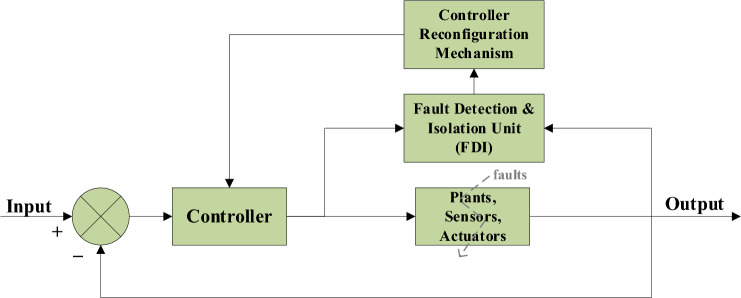
AFTCS Architecture^7^.

In the above equations, $x$ stands for the actual plant state, *u* for the control input, and *y* for the measured outputs. The nominal plant matrices are *A, B, C, and D*, $\overline{x }$ and $\overline{y}$ are estimated state value from observer and predicted model outputs respectively, and *L* is the observer gain. Equation 7 is the observer gain update, and Eq. 8 shows estimation-error dynamics. The AFTCS model uses these equations to dynamically modify the control input, ensuring that system performance is maintained even when a failure occurs. The fault in the system component will be determined by the residual. When the residual asymptotically converges to zero, the system is devoid of faults and stable. A component fault will be logged if the error surpasses the predefined threshold. The design of the FDI unit can employ several methodologies, including neural networks, fuzzy logic, and Kalman filters. The controller in AFTCS may detect many faults and functions in online mode. AFTCS architecture is shown in Fig. 1. ATCS suffers from the disadvantage of becoming slow and complex because of excessive calculations^5,6^.

The feedback control method is employed in a control system to achieve the desired response and stability. System stability is ensured by robust controllers that are unaffected by noise and parameter sensitivity^8^. The three predominant feedback control methods are derivative (D), integral (I), and proportional (P); the benefits of each action differ according to their alignment with the regulated application. The controller receives a set point and utilizes it along with sensor feedback to compute the error. These activities are then implemented, predominantly as P, PI, and PID^9^.

Redundancy, which is a vital element of FTCS, can be categorized into two forms: analytical and hardware. Hardware redundancy entails incorporating supplementary hardware to function as a backup component^10^. Conversely, hardware redundancy increases the system’s weight, cost, and size. In analytical redundancy, a virtual value for the sensor is generated by a software model of the component, which can be employed if the actual hardware malfunctions.

Different types of sensor faults can occur and can be controlled by FTCS systems, as also discussed in^11^. Sensor faults can include bias, i.e., a persistent offset resulting from calibration inaccuracies, scaling or gain errors, multiplicative inaccuracies leading to incorrect scaling, drift, a continuous deviation that may be linear or non-linear, and hard faults, i.e., fixed sensor output at a constant value, indicating failure. Furthermore, faults may be additive, introducing a constant or variable inaccuracy, or multiplicative, altering the output by a fault gain factor^12^. Moreover, an accumulation of factors, including simultaneous additive and multiplicative errors, may influence the precision and dependability of sensors. Significant operational challenges can arise from hard-over faults in centrifugal pump-based systems, which are often caused by bias in the speed or pressure sensors. Pump speed adjustments caused by these issues may be inaccurate, which in turn could affect the regulation of fluid levels and the reliability of the system. Uninterrupted operation and precise fault compensation depend on reliable fault isolation and detection systems.

### Pumping systems

Movement of fluids is the main function performed by the pumping system. The fluid is propelled through a rotating impeller using centrifugal force in the pump. Rotodynamic pumps and positive displacement pumps are the two primary groups according to their working principles, though there are numerous other ways to classify pumps. Centrifugal pumps are commonly used in industrial settings and are seen as a major component of the rotodynamic pumping system, even though positive displacement pumps are more efficient. Its limited flow rate and low-pressure head make it a relatively infrequent tool in large corporations^13^. Waide and Brunner^14^ classified centrifugal pumps as either single-stage or multi-stage, depending on the number of stages they contain. There is a wide range of uses for pumps.

Most pumps only have one stage; however, the high needs of heavy industries necessitate a big head^15^. The pump is compatible with both open-loop and closed-loop systems. A pump’s static head remains constant in a closed loop, but its dynamic head changes in response to the flow rate. Regardless of the value of the head, the flow rate will always be proportional to it in a pumping system. Suction and delivery reservoirs, pipe layouts, pipes for suction and discharge, and pump units are all part of the pumping system. A variable frequency drive, an alternating current supply, motors, and transformers make up the pump unit^16^. Keeping the motor speed under control is one method to save energy. Maintaining a steady speed requires 68% of the electrical energy consumed by flow loads^17^. In different applications, pumping units ensure the reliable and efficient transport of fluids. Maintaining the optimal pressure and flow rate is crucial in industrial systems because it ensures output and minimizes downtime.

### Importance of FTC for industrial systems

Many general industrial process control loops have problems with faulty sensors that lead to instability or a decrease in performance. A variety of domains have developed fault-tolerant control (FTC) solutions to continue the process when sensors fail. Several studies have examined FTC frameworks, both active and passive, that can handle sensor and actuator faults in different industrial settings^11^. Different studies have suggested FTC schemes tailored to diagnose and repair sensor faults in variable cycle engines^18^ and control the air–fuel ratio in internal combustion engines^19^.

Sensor faults are a well-studied and widely recognized problem with industrial pumping systems, and centrifugal pumps are also essential in many different sectors for fluid transportation. Using real-time sensor tracking, these devices allow one to maintain fluid levels. Malfunctioning sensors can produce overload, cavitation, or system shutdown resulting from control problems. This underlines how urgently reliable FTC systems are needed to keep systems running in the event of sensor failures for critical applications in industries.

### Literature review

Liquid level management is an important topic in industrial automation because of its relevance in controlling process and chemical industry operating variables, including pressure, temperature, and flow rates. Because of its affordability, simplicity, and reliability, PID controllers are commonly used in these types of applications. In liquid-level control, Poomani and Rameshwari^20^ demonstrated that an enhanced PID controller outperformed traditional Ziegler-Nichols PID controllers in terms of settling time and overshoot. The stability analysis relies on Lyapunov to control the flow of fluid in pipes, as demonstrated by Razvarz et al.^21^. As a result, vibration-induced system instability in the motor pumps was effectively decreased. Using PID controllers, which enabled reliable systems and precise height adjustment, was highlighted by Getu^22^. To highlight the importance of controllers in maintaining process efficiency, Fellaini and Gabaj^23^ studied the function of PID controllers in regulating water flow into tanks and preventing overflow.

Though controllers are widely used, the growing complexity of industrial systems demands FTC solutions to manage sensor faults and ensure dependable operation. Problems like unanticipated abnormalities or component malfunctions might seriously compromise or even cause catastrophe for system performance. Kalman filters, state observers, adaptive observers, and unknown input observers are among the approaches for estimating errors^7^. These approaches improve system dependability by increasing the accuracy of fault detection and allowing quick action. Through proper fault estimation, the FTC technique guarantees system stability by lowering the effect of failures. Advanced FTC algorithms will enable the hydraulic system to run even in the situation of unforeseen and unexpected malfunctions. These developments have made fault-tolerant systems indispensable parts of industrial control system architecture.

Recent studies confirm that sensor-fault FDI/FTC is relevant and useful for process and liquid-level systems. Amor et al. demonstrated the feasibility of observer-based techniques in water-level plants by developing an FTC system for a three-tank process. The system utilized a bank of high-gain observers for sensor-fault FDI and controller reconfiguration for tank benchmarks^24^. Complementing observer methods, Ortega et al. demonstrated the efficacy of data-driven FDI directly in level-control hardware by reporting unsupervised defect detection on a controlled conical tank^25^. Kazemi et al. worked on an approach to process control, emphasizing the importance of clear and understandable decision criteria in dynamic operating environments by creating time-varying process FDI/Isolation processes^26^. Workneh et al. demonstrated the necessity for deployable, comparable baselines in level systems by introducing a liquid-level benchmark platform tailored for testing model-based fault diagnostics in interconnected tanks^27^. Our study provides a lightweight and interpretable baseline for centrifugal-pump level control that can be easily extended to more complex observers or data-driven schemes. This is important because these works collectively demonstrate an increasing interest in practical, testbed-driven FDI/FTC for processes like tanks and pumps.

The study’s overarching goal is to find out how well the FTC algorithm works in a variety of contexts. To ensure that industrial combustion engines continue to operate reliably in the case of failure, researchers have also looked into active FTC^19^. In literature, studies have also concentrated on applying FTC techniques to increase the dependability of pump-operated electrohydraulic systems by reducing performance drop susceptibility to malfunction^28,29^. The growing complexity of modern control systems necessitates the development of new FTC methods to guarantee safety in many technical applications and to raise fault tolerance^30^. The continuous improvement of FTC techniques is driving advancements in automation^31^.

This paper offers the following contributions: This study addresses FTC for a centrifugal pump level control system, emphasizing fault detection and tolerance for three critical system sensors:

- Level Sensor: Determines the level of liquid in tank and delivers immediate feedback to facilitate the maintenance of the specified setpoint.
- Centrifugal Pump Speed Sensor: Tracks the pump’s rotational speed to maintain consistent fluid flow and detect any discrepancies resulting from malfunctions.
- Discharge pressure sensor: A centrifugal pump discharge pressure sensor monitors the pump’s discharge pressure to identify anomalous pressure variations and maintain optimal flow conditions.

Implementing the level sensor to keep the tank level is the key objective. Despite not being directly dependent on them, two other sensors are incorporated to validate the system’s reaction in this study. In real-world industrial pumping systems, where pump speed and discharge pressure are constantly monitored for performance evaluation, safety supervision, and maintenance diagnostics, this concept will prove to be extremely essential. Hence, sensor readings have a crucial supervisory and supporting function in the overall system architecture. This study establishes the FTC method to address sensor faults through the application of analytical redundancy and projected values in the event of sensor failures. It is considered that there will be faults in any one sensor at a time. The generated FTC model demonstrates the effectiveness of the suggested method in maintaining control performance under challenging circumstances by ensuring system stability and reliable operation despite sensor faults.

The contents in paper are further organized into three sections. Section 2 discusses the system description, assumptions made, and research methodology overview, whereas Section 3 details the results and discussions. The study’s conclusion is summarized in the last section.

## Research methodology

**Fig. 2:**
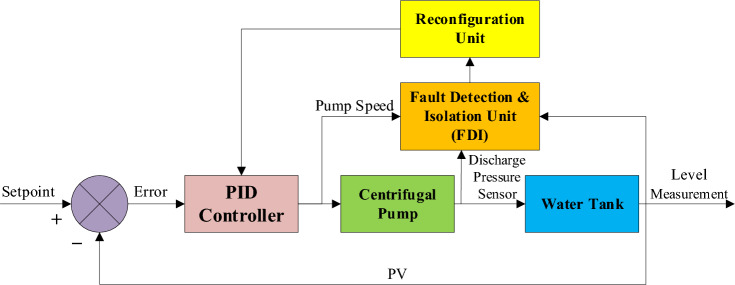
System block diagram.

This study develops an AFTCS for a centrifugal pump-based water tank level control system using MATLAB and Simulink. The system includes an FDI unit that calculates sensor values in the case of a fault in order to increase overall dependability. Figure 2 shows a block diagram that visually represents the entire system architecture.

**Fig. 3:**
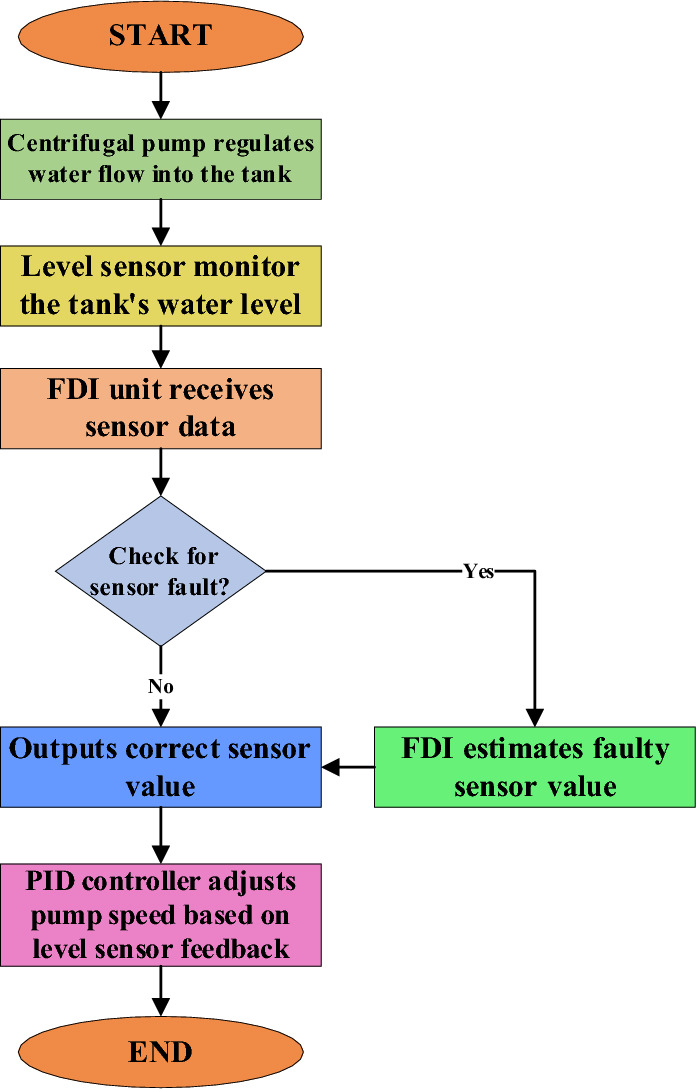
Flowchart of the proposed study.

A fault-tolerant, closed-loop level-control system is developed as shown in the block diagram. PID uses data from a level sensor at the tank’s output to keep the water level at 1.4 m. In the event of a fault, the PID controller controls the pump to run at a required speed after comparing the setpoint with the measured process variable from feedback. Sensors monitor the tank water level, centrifugal pump speed, and discharge pressure. These sensor signals are received by the FDI unit. An observer based on the statistical linear regression technique is utilized by the AFTCS FDI unit. It calculates the difference between each measurement and calculates residuals. The estimation unit is also part of the FDI block, which estimates faulty sensor values using regression. The PID loop continues operating after the Reconfiguration unit emits a faulty value and substitutes the inaccurate measurement with its estimation. For instance, if a fault occurs in the level sensor, it estimates the water level using data from the centrifugal pump speed and discharge pressure sensors. The FDI unit makes sure the system keeps running even if a level sensor fails. This estimation makes use of the multiple linear regression technique to successfully avoid system shutdown and keep operations stable in the case of sensor failures. Figure 3 also shows the associated flowchart that details the complete proposed procedure step-by-step.

This research employs the single-fault assumption, which states that at any given moment, a single sensor, i.e., level, pressure, or speed, could have a fault, meaning the sensor’s output remains at zero in case of a fault. Due to this assumption, real-time implementation with less processing complexity and dependable fault isolation can be both made achievable. Under healthy circumstances, sensor readings remain unchanged. Also, the study only employs a stuck-at-zero fault as a representative hard sensor fault because it causes immediate degradation of closed-loop performance and full loss of measurement information. These types of faults are widely employed in FTC validation to test how well fault detection and signal reconfiguration algorithms perform in undesirable conditions.

The study is carried out using an already existing MathWorks model, which consists of a jet pump, surface-mounted centrifugal pump, and tank^32^. A jet pump extracts water from the well (water source) in the MATLAB model, and then a centrifugal pump transfers the water to the tank. Centrifugal pump parameters were kept the same as they were in the original MATLAB model and were not changed for this study. According to Fox et al.^33^, the Bernoulli head (H) determines the energy increase produced by the pump in the case of adiabatic, isothermal, and steady-state flow of an incompressible fluid. A centrifugal pump’s total head (H) is determined by:

12 $$H=\frac{\left({P}_{Discharge}-{P}_{Intake}\right)}{\left(\rho g\right)}+\frac{\left({v}_{Discharge}^{2}-{v}_{Intake}^{2}\right)}{\left(2g\right)}+\left({z}_{Discharge}-{z}_{Intake}\right)$$

where ${P}_{Discharge}$ is the pressure at pump discharge, ${P}_{Intake}$ is the pressure at pump intake, $\rho$ denotes the density of the fluid, the gravitational constant is denoted by $g$, ${z}_{Discharge}$ is the elevation at discharge point, and ${z}_{Intake}$ is the elevation at intake point. H and Δp, the pressure rise, are practically proportional, hence given by^34^:

13 $$H=\frac{\left({P}_{Discharge}-{P}_{Intake}\right)}{\left(\rho g\right)}$$

According to Gülich^35^, centrifugal pumps are susceptible to three types of losses: shock losses, leakage losses, and hydraulic losses. The efficiency (η) equation shows that the power consumed by the pump is always greater than the power effectively delivered to the fluid.

14 $$\eta =\frac{\rho gHQ}{\left(\omega T\right)}$$

where in the above equation, *Q* belongs to the volumetric flow rate, $\rho$ denotes the density of the fluid, *v* is the average velocity of fluid flow, *ω* refers to the angular velocity of the shaft, *T* is the shaft torque, and $g$ is the gravitational constant. Fluid dynamics impact the centrifugal pump-based tank’s level control system, and Bernoulli’s equation describes the exit flow^36^:

15 $$P+\left(\frac{1}{2}\right)\rho {v}^{2}+\rho gh=constant$$

A distance of 36 feet separates the water level from the centrifugal pump. After making any required adjustments to the model to fit the AFTCS design, the findings were carefully analyzed. Initially, a PID controller is used to operate the system. In the existing operating situation, this system uses a PID controller to maintain a consistent liquid level in the tank. According to the following control law, as also given in^21^:

16 $$u\left(t\right)={K}_{P}e\left(t\right)+{K}_{I}\int e\left(t\right)dt+{K}_{D}\left(de\left(t\right)/dt\right)$$

17 $$u(t) ={K}_{P}X+{K}_{I}\int Xd\tau +{K}_{D}\dot{X}$$

18 $$X ={h}_{desired}- h\left(t\right)=e(t)$$

In the above equations, ${K}_{P}$,${K}_{I}$, and ${K}_{D}$ are proportional, integral, and derivative gains of the controller, respectively. Whereas, u(t) is the controller output signal, e(t) is the error between the desired and actual tank levels. ${h}_{desired}$ is the reference tank level (set point), and *h* is the actual tank level at time t.

This causes the following rate of change in the tank level:

19 $$\dot{h}\left(t\right)=\frac{1}{A}\left( u(t) -{Q}_{out}h\right)$$

Substituting Eq. (17) into (19) yields:

20 $$\dot{h}\left(t\right)=-\dot{X}(t)=\frac{1}{A}\left(({K}_{p}X+{K}_{i}\int Xd\tau +{K}_{d}\dot{X}){-Q}_{out}h\right)$$

21 $$\mathrm{u}\left(\mathrm{t}\right)={K}_{p}X+{K}_{d}\dot{X}+\vartheta$$

where

22 $$\vartheta ={K}_{i}\int X(\tau )d\tau ,\vartheta \left(0\right)=0$$

Also,

23 $$\dot{{\vartheta }}={K}_{i}X$$

### ***Proof***

Linearized PID control error dynamics indicate the controller shaping the tank’s level about the operating point.

24 $$\frac{d}{dt}\left[\begin{array}{c}\vartheta \\ X\end{array}\right]=\left[\begin{array}{cc}0& {K}_{i}\\ -\frac{1}{A+{K}_{d}}& -\frac{{K}_{p}+c}{A+{K}_{d}}\end{array}\right] \left[\begin{array}{c}\vartheta \\ X\end{array}\right]$$

where A is the cross-sectional area of the tank, and c = ${Q}_{out}$.The positive-definite quadratic Lyapunov function on the PID state x is used to assess stability.

25 $$V\left(\vartheta ,X\right)=\frac{1}{2}{x}^{T}Px=\frac{1}{2}\left({\frac{{\vartheta }^{2}}{{K}_{i}(A+{K}_{d})}+X}^{2}\right)$$

From (25), since *V* is zero only at equilibrium, and *V* is negative semidefinite and never increases.

26 $$\dot{V}=\left(-\frac{1}{A+{K}_{d}}\vartheta -\frac{{K}_{p}+c}{A+{K}_{d}}X\right) X=-\frac{{K}_{p}+c}{A+{K}_{d}} {X}^{2}\le 0$$

27 $$\dot{V}=0\Rightarrow (\vartheta , X)=(\mathrm{0,0})$$

Assuming a locally rising outflow at the operational point and positive controller gains, the Lyapunov argument demonstrates that the integrator states and error converge to zero under normal conditions. Consequently, the PID controller ensures that the regulatory equilibrium is locally stable, even to an exponential degree.

Ensuring efficient operation, decreasing energy consumption, preventing overflow or damage to pumps, and maintaining the exact tank level are essential for many industrial applications. Industries such as water treatment facilities, chemical processing, and power generation could benefit from this research since they all rely on safe and effective fluid level control. Additionally, the performance of fault-tolerant pump speed and discharge pressure sensors is also evaluated by extending the suggested AFTCS. The concept’s adaptability is shown by validating these other two sensors’ fault-tolerant performance, even though they do not directly control the system. The study also investigates the FTC’s performance with speed and pressure sensors in addition to its primary function of handling level sensor faults. The pressure and speed sensor outputs from the FDI unit could be useful for monitoring or other uses in industrial systems, even though they aren’t used for system control in this study. It is demonstrated that the system is suitable for fault identification and that it can withstand the failure of a single sensor at a time by accurately computing its values using data from the other two sensors. Accommodating only one sensor fault at a time is the limitation of this study. Also, this study is conducted considering water as a fluid. Systematic simulation of sensor failure demonstrates the efficacy of the suggested AFTCS in enhancing system reliability in fault scenarios.

### Multiple linear regression technique

A very common and basic statistical method for figuring out the relationship between dependent and independent variables is linear regression, which creates a linear model. The resulting relationship is of the general type:

28 $$y = \beta x+\varepsilon$$

where the values of the dependent and independent variables are represented by the vectors "y" and "x", and the error or noise term is denoted by $"\varepsilon$". Values of partial derivatives of dependent variables concerning different independent variables are represented by the symbol "β." This method is used to find out how the variables in the data set are related to each other, and then the model can be used to predict what the numbers will be in the future. This method can also be used to assess how well the model fits the data that was collected^37^. Consequently, this method is employed for AFTCS observer design since it can be estimated from the observation data set of the model. In comparison to other FDI methods, linear regression is computationally easy and doesn’t need system modeling parameters; by applying it to the data, one may generate linear functions of variables that can be instantly incorporated into the FDI, which is its main advantage. On the other hand, because most physical processes are nonlinear, their accuracy is often only valid within a small linear range, and it relies significantly on high-quality data^38^.

The multiple linear regression (MLR) model expands upon this by taking into consideration many independent variables. In numerous technical and scientific contexts, this statistical method is employed to capture detailed input–output connections. Finding an approximate linear function that shows the relationship between the dependent variable and multiple independent factors is the main objective of MLR^39^. Here is a way to express the MLR regression equation that maintains its generalizability:

29 $$y = {\beta }_{1}{x}_{1 }+ {\beta }_{i}{x}_{i}+\cdots +\varepsilon$$

where ${x}_{i}$ is the ith number of independent variables, and *y* denotes the output, which is to be estimated. β is the polynomial coefficient. It offers a strong foundation for estimating dependent variables from several related factors, making this model especially effective in situations where FTC depends on multiple sensor inputs for system stability and estimation. In real-time control settings, an estimator based on MLR is primarily used since it is easy to implement and requires less processing complexity. Unlike model-based observers, which require precise system models, noise statistics, and online matrix operations, the proposed MLR estimator relies on straightforward algebraic computations, making commercially available controllers suitable for implementation. The fundamental purpose of the estimator is not high-fidelity modelling but rather the successful reconfiguration of faulty sensor data in order to keep the closed-loop system operational with low processing cost.

## Results and discussion

**Fig. 4:**
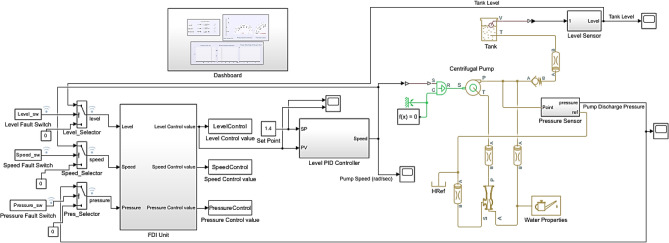
Complete system model.

The entire system model with the implementation of the AFTCS for the water tank level control in the MATLAB and Simulink environment, including the FDI unit, is given in Fig. 4. All components and their working inside the system are shown in this figure.

As discussed in the previous section, for the system to regulate the tank water level as effectively as possible, the gains of the PID controller and the settings of the centrifugal pump are important. Table 1 presents the results of the careful tuning of the PID controller gains, which ensured effective and consistent regulation of the centrifugal pump’s speed to get the desired fluid level in the tank, and transient response in faulty conditions. By improving the pump’s reaction to changes in water level, the response guarantees accurate and robust control of the system. Using time-domain performance objectives as a basis, the robust PID controller gains were obtained through an iterative tuning procedure. The initial gain levels were carefully selected to provide stable operation and a suitable transient response. Then, to tune the system and guarantee consistent closed-loop performance using the MATLAB PID tuning interface.

**Table 1:** PID controller gain values and transient response in faulty conditions.

Parameters	Values
Gain Values	P = 70k, I = 21, D = 1k
Rise time	6.5299 s
Settling time	7.9952 s
Settling minimum	1.2882
Settling maximum	1.3999

**Fig. 5:**
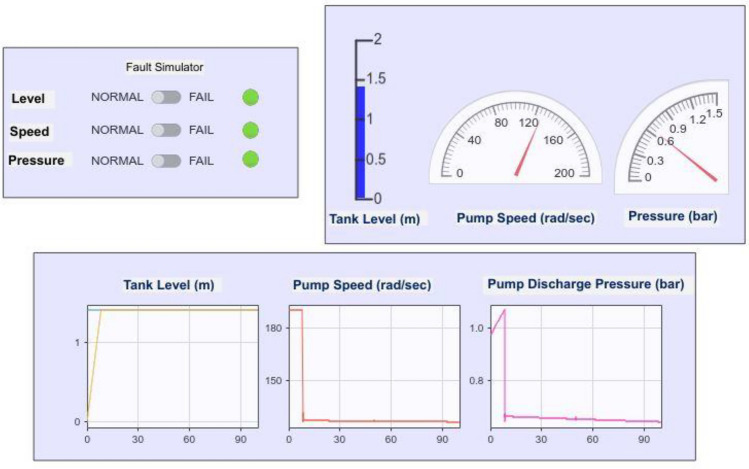
FIU and Dashboard of Scopes.

The dashboard is shown in Fig. 5, which also includes a fault injection unit (FIU) and is used to analyze the effects of faults introduced into each sensor separately. To ensure that the control algorithm is robust, this feature of fault injection replicates sensor failures. The dashboard also has gauges that display sensor data in real time and provide a clear view of the system’s behavior.

**Fig. 6:**
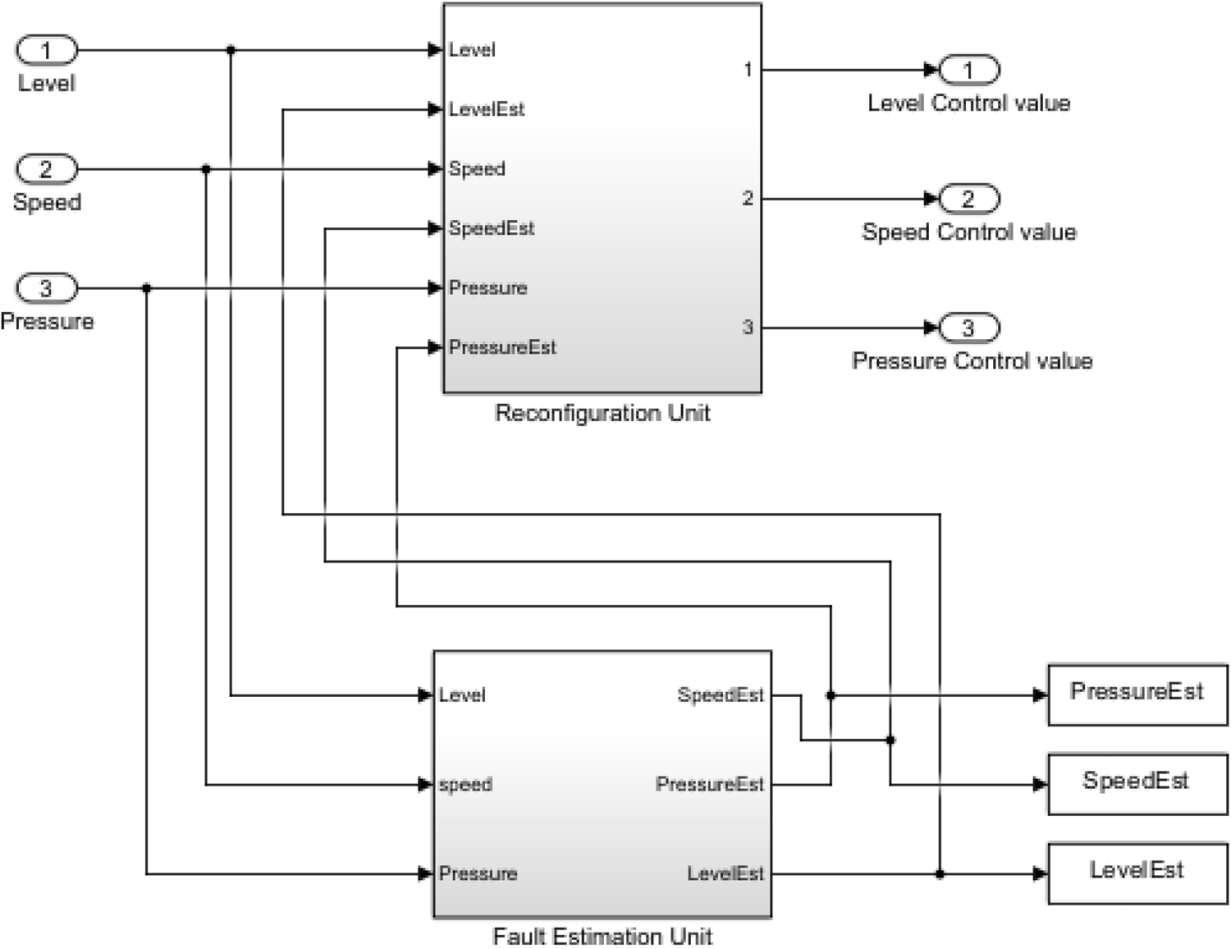
Internal Architecture of FDI unit.

To maintain continuous system functioning while identifying and isolating issues with the level, pressure, and speed sensors, the FDI block is important. The FDI block’s internal architecture consists of the reconfiguration block and the estimation block, as shown in Fig. 6.

In MATLAB, an MLR technique is applied to develop the fault estimation unit with data from model simulations of all sensors behaving normally. The MLR model that has been employed based on data from healthy sensors is utilized by the estimation block to estimate the faulty parameter from the values of the other connected sensors.

The estimated tank level (y) can be found by substituting the centrifugal pump speed (x₁) and discharge pressure (x₂) into the regression equation:

30 $$\mathrm{y}=3.81-0.026065{x}_{1}+1.38{x}_{2}$$

Similarly, regression equations for speed estimation and pressure estimation, respectively, are calculated as:

31 $$y=81.027-18.962{x}_{1}+115.05{x}_{2}$$

32 $$y=-0.288+0.0066019{x}_{1}+0.057797{x}_{2}$$

The second subpart of the FDI unit, the reconfiguration block, checks residual value at regular intervals for changes brought about by sensor failures, typically as:

33 $$r\left( t \right) = y(t) - \hat{y}(t)$$

where y(t) denotes real sensor output, $\widehat{y}$(t) represents estimated sensor output by the estimation unit, and r(t) represents calculated residual. If r(t) ≥ threshold value, the reconfiguration unit will give a correct sensor output from the estimation unit instead of the original faulty sensor value. The PID controller receives the estimated value of the tank level from the estimation block instead of the faulty sensor value when the residual is higher than the predefined threshold. This ensures that the system continues to operate.

**Fig. 7:**
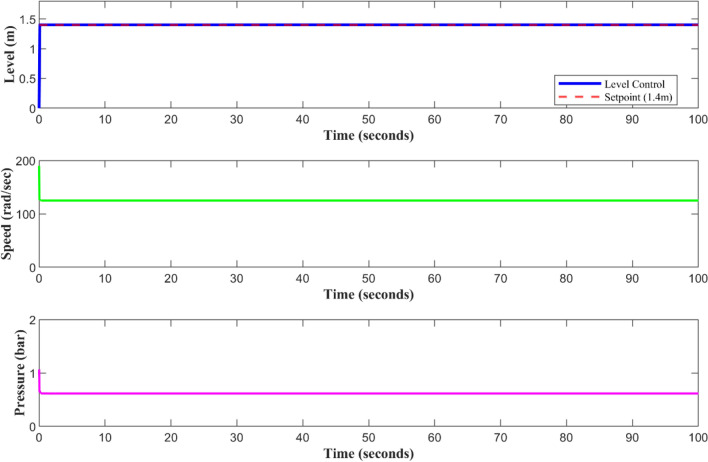
All sensors are working in a healthy condition.

In Fig. 7, it can be seen how each sensor should work when healthy and how the level sensor keeps the water level at the 1.4 m setpoint. In a nominal control loop, the pump speed and discharge pressure remain constant, the tank level tracks the reference value without overshooting, and the operation is performed. Providing the FDI residual stays below the detection threshold and the reconfiguration logic is not being used, the AFTCS-PID response is the same as the baseline PID under normal conditions after the controller transmits the precise measured level signal.

**Fig. 8:**
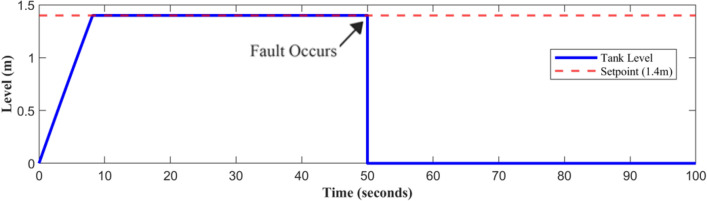
Level sensor behavior in case of fault without FTC.

**Fig. 9:**
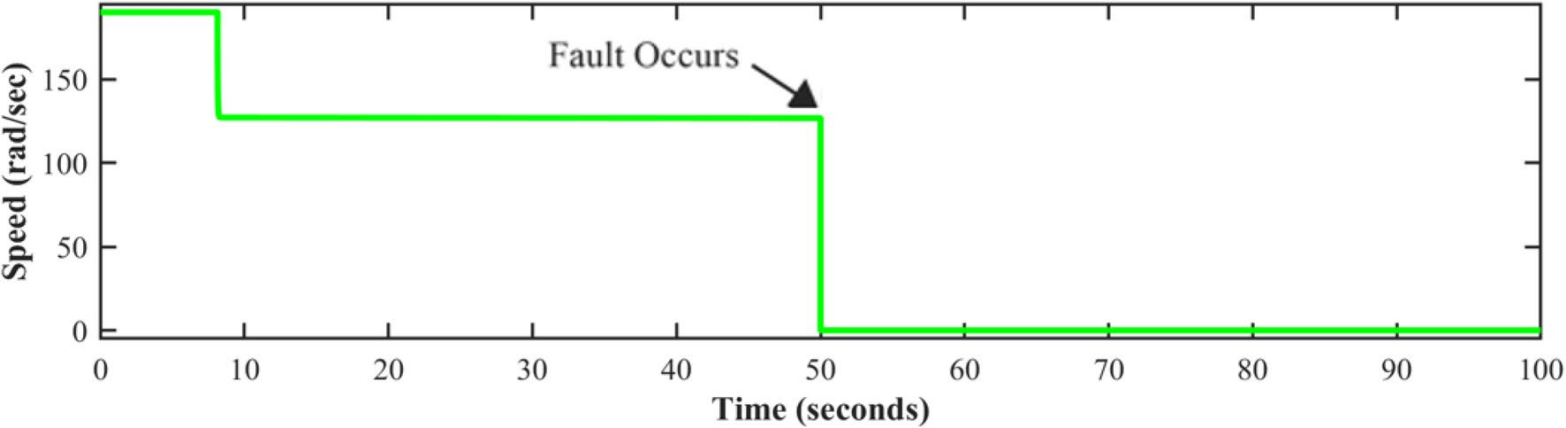
Speed sensor behavior in case of fault without FTC.

**Fig. 10:**
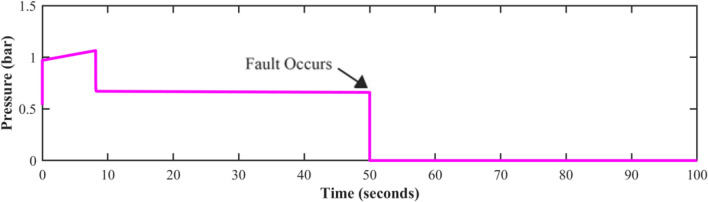
Discharge pressure sensor behavior, in the presence of a fault without FTC.

Fault-tolerant level sensors are essential in pumping systems due to the significant operational challenges posed by sensor failures. A level sensor failure would lead the controller to acquire wrong data, which would cause the pump to function incorrectly. Either too much or too little water could cause damage to downstream processes or equipment. In industrial settings, unidentified sensor failures can create system interruptions, contribute to greater maintenance costs, and even endanger workers. Figures 8, 9, and 10 show the behavior of the system at time t = 50 s. The potential shutdown induced by a sensor stuck at zero failure, which would force all measured channels to zero, in the absence of FTC, highlights the necessity of this approach. Furthermore, ignoring sensor faults might compromise the overall efficacy and dependability of automated systems. Fault-tolerant strategies are designed to keep systems running smoothly, enhance accuracy, and prevent costly malfunctions.

**Fig. 11:**
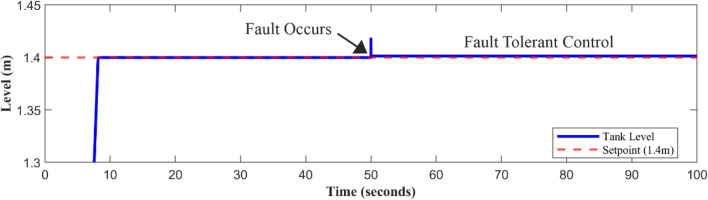
Level Sensor FTC.

**Fig. 12:**
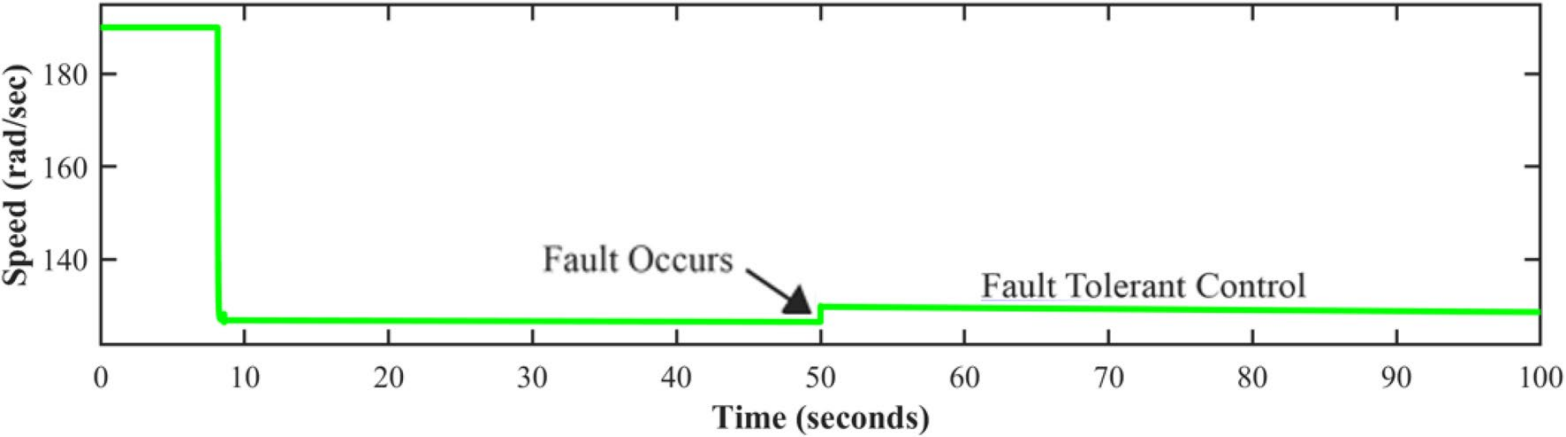
FTC applied to the pump speed sensor.

**Fig. 13:**
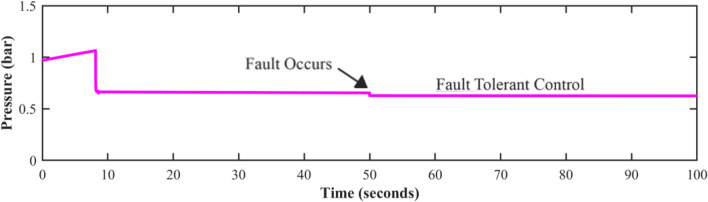
Pressure sensor behavior in case of fault.

The AFTCS can prevent system shutdown by substituting inaccurate sensor data with correct estimations, as shown in Figs. 11, 12, and 13, demonstrating the system’s reliability in controlling sensor failures. Fast and accurate measurements are quickly produced by the FDI reconfiguration even after fault injection. It is noted that the level sensor has a recovery time of around 2 ms, whereas the pressure and speed signals take approximately 40 ms for them to get back to their original values; hence, closed-loop control is unaffected.

The fault is induced in all sensors one by one at 50 s (as marked), and it is also shown by figures that the system immediately shifts to estimated sensor values being fed by the FDI unit in case of fault occurrence. Although the centrifugal pump discharge pressure sensor and speed sensor are monitored, and the fault tolerance concept is also applied to ensure system operation, it does not directly affect the level control loop in this system. Analytical redundancy’s predicted values can help in the detection of sensor faults, ensuring the system’s integrity for use in the future. This work employs the FTC approach to keep tank-level management going in case any of the sensors fail. Meanwhile, the pressure and speed sensors of the pump give more data for validating the system and making improvements.

## Comparison with existing studies

Overall, by contrasting the system behavior under healthy sensor conditions with the same controller but with sensor faults, the performance degradation without FTC and the recovery attained by the proposed FDI-based reconfiguration, all results are illustrated in detail in the previous section. To the best of their knowledge, the authors did not find any research paper or work that has worked on the same model and the proposed FTC approach. In this study, a novel technique for improving the reliability and efficacy of systems that are based on centrifugal pumps is presented. It is possible to compare the findings of this investigation with those of the initial MATLAB model, which did not incorporate essential components such as the FTC algorithms, sensors, or the PID controller. Control and fault-tolerant parameters were not present in the system that was previously in place. The special combination of these cutting-edge control methods that are presented in this study offers a solution that is more reliable for industrial systems. This study not only enhances the performance of the system but also provides the framework for future efforts to deploy intelligent and fault-tolerant approaches in industrial settings. This will ensure that there will be improvements in operational effectiveness that are practically applicable. By influencing the development and implementation of FTC systems, this research has the potential to improve the flexibility, dependability, and capability of centrifugal pumps to continue working even if a sensor fault occurs. Table 2 compares this study with some recent research on tank/pump level systems conducted using FTC or FDI.

**Table 2:** Comparison with some other studies.

Refs	Application	Applied Technique	Addressed faults	Strengths	Limitations
^24^	Three-tank system	Observer-based for FDI + active reconfiguration	Sensor faults	Formal FDI and FTC; validation of the benchmark system	Greater design complexity; precise models and observer adjustments are needed
^40^	Heated two-tank system	Multiway principal component analysis	Leakage faults, Blockage	Manages mode shifts and transients; experimental	No FTC loop
^41^	Three-tank nonlinear system	Differential flatness-based FDI	Sensors and actuators (multiplicative faults)	Strong sensitivity analysis, experimental proof is also provided	Effort on modeling and flatness; no fault reconfiguration
^27^	Three-tank liquid-level control system	First-principles modeling + least-squares system identification	Sensor and actuator faults (experimental data)	Real experimental benchmark; open-access fault data; suitable for validating model-based FDI algorithms	Focused on diagnosis only; no fault-tolerant control or closed-loop compensation
This study	Centrifugal pump-based tank water level control	Fault detection + Regression-based estimator	Sensor Faults	Low complexity, easy implementation on commercially available controllers, prevents system shutdown	Assume only one type of fault at a time, simulation only

## Conclusion

The FTC method for a water level control system based on a centrifugal pump is presented in this research. This paper developed and applied a reliable FTC technique for a centrifugal pump-based system to overcome sensor faults. The sensors considered were the tank level sensor, pump discharge pressure sensor, and centrifugal pump speed sensor. To control the pump speed and ensure a sufficient water flow, a PID controller was incorporated into the system to maintain the target tank level. Equipped with an FDI unit, the level sensor may estimate its values depending on data from the remaining two sensors in case of faults. Similarly, if any one of the sensors became faulty, the system continued to work reliably because of an FDI subsystem and a linear regression model for estimation. The suggested method successfully mitigates the effects of common sensor errors, according to simulation results. The necessity to apply FTC techniques to industrial systems to improve operational efficiency and reliability was emphasized in this research.

Future studies should concentrate on extending the proposed approach to concurrent multi-sensor failure scenarios and carrying out experimental validation on hardware platforms. Future hardware-in-the-loop validation work may focus on integrating the controller and sensor interfaces into hardware to assess the impact of delays, sampling, and real-time execution. Future research should also work on the aspect of actuator-level faults, sensor noise, and communication delays, to show real-time performance and practical deployability beyond simulation. To improve fault detection and adaptive control, future studies can concentrate on expanding the model to incorporate advanced methods and can also address different fault types, such as drift, bias, etc. This work has the potential to inspire future research directions, including the development of advanced FTC schemes for industrial systems with several sensors. The robustness and predicted precision of real-time systems’ FDI by the application of machine learning methods can also be enhanced. Moreover, investigation of FTC in more intricate systems, like mechanical systems that are networked or multi-phase pumps, is also a potential future research direction.

## Data Availability

No datasets were generated or analyzed during the current study.

## Abbreviations

AFTCS: Active fault-tolerant control systems
FTC: Fault tolerant control
FTCS: Fault tolerant control systems
FIU: Fault injection init
FDI: Fault detection and isolation
MLR: Multiple linear regression
PID: Proportional integral derivative
$x$: Actual plant state
*u*: Control input
*y*: Measured output
*L*: Observer gain
${P}_{Discharge}$: Pressure at pump discharge
${P}_{Intake}$: Pressure at pump intake
$\rho$: Density of the fluid
$$g$$: Gravitational constant
${z}_{Discharge}$: Elevation at the discharge point
${z}_{Intake}$: Elevation at the intake point
*Q*: Volumetric flow rate
*v*: Average velocity of fluid flow
*T*: Shaft torque
${h}_{desired}$: Reference tank level (set point)
*h*: Actual tank level at time t
*e(t)*: Error between the desired and actual tank level
*u(t)*: Controller output
*r(t)*: Residual
$${K}_{P}$$: Proportional gain
$${K}_{I}$$: Integral gain
$${K}_{D}$$: Derivative gain
